# Can Slow-Motion Footage of Forehand Strokes Be Used to Immediately Improve Anticipatory Judgments in Tennis?

**DOI:** 10.3389/fpsyg.2018.01830

**Published:** 2018-10-04

**Authors:** Kazunobu Fukuhara, Tomoko Maruyama, Hirofumi Ida, Takahiro Ogata, Bumpei Sato, Motonobu Ishii, Takahiro Higuchi

**Affiliations:** ^1^Department of Health Promotion Science, Graduate School of Human Health Science, Tokyo Metropolitan University, Hachioji, Japan; ^2^Department of Sports and Health Management, Jobu University, Isesaki, Japan; ^3^Department of Sport and Medical Science, Teikyo University, Hachioji, Japan; ^4^Graduate School of Health and Sport Science, Nippon Sport Science University, Kanagawa, Japan; ^5^Department of Human System Science, Tokyo Institute of Technology, Tokyo, Japan

**Keywords:** anticipation, motion recognition, computer graphics, expertise, sport

## Abstract

Slow-motion footage of sports actions is widely used as a visual learning tool in observing the dynamic motor behaviors of athletes. Recent studies on action observation have reported that extending the observation time in slow-motion footage provides benefits of understanding the intention of an opponent’s action, at least when observing rapid movements. As such, the use of slow-motion footage may have the potential to improve the anticipatory judgments of an opponent’s action outcome without training (or feedback). To verify this possibility, we examined the effects of the replay speed of slow-motion footage on the anticipatory judgments of shot directions and recognition of kinematic positions of opponents’ forehand strokes in tennis. Nine skilled and nine novice tennis players were asked to anticipate the direction of their opponent’s shots (left or right) and then attempted to recognize proximal (trunk center) and distal (ball) kinematic positions. Computer graphic animations of forehand strokes were used as visual stimuli, which were presented at four different replay speeds (normal, three-quarter, half, and quarter speeds). We failed to show the immediate effect of the use of slow-motion footage on the anticipatory performance of the skilled and novice players, although the anticipatory performance of the skilled players was superior to that of the novice players. Instead, we found an effect of the use of slow-motion footage in terms of promoting recognition of important kinematic cues (trunk center) for effective anticipation by skilled players. Moreover, no significant correlations were observed between the anticipatory judgments and motion recognition in all experimental conditions. These results suggest that even if the use of slow-motion footage enhances the recognition of key kinematic cues, it may not immediately improve anticipatory judgments in tennis.

## Introduction

Slow-motion footage of sports actions is widely used as a visual learning tool in observing complex and quick motor behaviors by athletes, such as a golfer’s swing movement and a tennis player’s forehand stroke (Williams et al., 2002; Wilson, 2008). Recent studies (Moriuchi et al., 2014; Moriuchi et al., 2017) on action observation have reported that extension of the observation time in slow-motion footage provides benefits of understanding the intention of an opponent’s action, at least when observing rapid movements. Moriuchi et al. (2014) examined how speeds of observed actions affected the excitability of the primary motor cortex (M1). The size of motor-evoked potentials of the hand muscles was induced by transcranial magnetic stimulation (TMS) when participants observed a video footage of an individual catching a ball at three different replay speeds (normal, half, and quarter speeds). The results showed that the excitability of the M1 was higher when the observed action was at low-speed replays (half and quarter speeds) than at normal-speed replays. More recently, Moriuchi et al. (2017) reported that the same effects were confirmed only when viewing the low-speed replay video of rapid movements (i.e., catching a ball); such effects were not confirmed when viewing slow movements (i.e., reaching for and lifting a ball). The authors explained that the benefit of using slow-motion footage is likely to be obtained only for rapid movements, in which the components of observed actions would not be visible at normal speed. In other words, there seems to be no benefit in using slow-motion footage for slow movements, for which observers could recognize the components of actions even at normal speed. As such, the use of slow-motion footage should lead to the activation of the action observation network (AON), allowing understanding of an opponent’s action intention (Rizzolatti et al., 2001) when applied to rapid movements.

The present study was designed to investigate whether the use of slow-motion footage of forehand strokes can immediately improve anticipatory judgments of shot directions and recognition of kinematic positions of opponents’ forehand strokes in tennis. The ability to anticipate the direction of an opponent’s forthcoming shots is important to return a shot successfully in racket sports, such as badminton and tennis. The forehand stroke in tennis is a rapid movement; therefore, the use of slow-motion footage should lead to the activation of the AON. Considering that skilled anticipatory judgments are underpinned by the detection of key kinematic cues from an opponent’s movements (Jones and Miles, 1978; Shim et al., 2005; Abernethy and Zawi, 2007; Jackson and Mogan, 2007; Williams et al., 2009; Ida et al., 2011a,b; Fukuhara et al., 2017), the prolonged time afforded to detect key kinematic cues from an opponent’s movements would lead to better anticipatory performance.

To date, no study has investigated the effects of the use of slow-motion footage on anticipatory judgments in racket sports. Moreover, two studies did not support the effectiveness of the use of slow-motion footage on anticipatory judgments in other types of rapid movements (Lorains et al., 2013; Uchida et al., 2014). Uchida et al. (2014) showed that in the anticipation task of free throw shot success in basketball, the anticipation accuracy of experienced players decreased when they viewed the slow-speed motion condition (half speed). The authors suggested that the reason for the decrement in anticipatory performance with the use of the slow-speed video was derived from the “mismatch” between the temporal information acquired through experience and the stimulus’s temporal information (Barclay et al., 1978). Lorains et al. (2013) also found no improvement in a video-based decision-making task in Australian football under the slow-speed motion condition (three-quarter speed).

Herein, we examined the anticipatory judgments of shot directions and recognition of opponents’ kinematic positions (errors between subjective evaluation findings and measured values) when skilled and novice tennis players viewed slow-motion footage of tennis forehand strokes at four different replay speeds (normal, three-quarter, half, and quarter speeds). Based on the findings of the two studies that did not support the effectiveness of the use of slow-motion footage (Lorains et al., 2013; Uchida et al., 2014), we speculated that the replay speed could affect the benefit of the use of slow-motion footage. Therefore, we adopted four replay speeds, two of which were the same as those in the studies of Uchida et al. (2014) and Lorains et al. (2013).

We also evaluated the recognition of kinematic positions using the visual analog scale (VAS). We speculated that the benefit of using slow-motion footage may come in part from the prolonged time available for detecting key kinematic cues from an opponent’s movements. If this is the case, then the recognition of the opponent’s kinematic position would also be improved when slow-speed footage is used. Therefore, we tested this possibility with this recognition performance.

We hypothesized that the correct responses and recognition errors in both skilled and novice players would be improved with the decline in replay speeds. Moreover, if enhancing the recognition of key kinematic cues improves anticipatory judgment, then it was hypothesized that there would be a strong correlation between both performances. We also hypothesized that skilled players would outperform their novice counterparts in anticipating shot directions based on the findings of previous studies regarding anticipation in tennis (Shim et al., 2005; Williams et al., 2009; Fukuhara et al., 2017).

## Materials and Methods

### Participants

Nine skilled tennis players (*M_age_* = 19.8 ± 1.5 years, 12.2 ± 2.2 years of tennis experience) and 9 novice counterparts (*M*_age_ = 22.2 ± 4.7 years) participated in this study. Skilled players were on a university tennis team that had played in national tournaments. Additionally, this team had won in all-Japan intercollegiate tournaments in 2016. Novices had played tennis at least once in physical education class but did not play regularly. The experimental protocol was approved by the institutional ethics committee of Tokyo Metropolitan University (authorization number H27–36). The tenets of the Declaration of Helsinki were followed. All participants gave written informed consent prior to participation. None of the participants had previous experience with the experimental task or procedure.

### Visual Stimuli

We adopted computer graphic (CG) animations as visual stimuli to accurately evaluate recognition errors between the VAS scores and the original coordinate position output from motion capture data. We used CG animations of forehand shots to test the evaluation validity for anticipatory judgment of shot direction (Fukuhara et al., 2009; Fukuhara et al., 2017). First, forehand stroke shots by a professional tennis player (22 years old, 11 years of tennis experience, and ranked in the top 30 in Japan) were recorded on the tennis court using three-dimensional motion capture cameras (Hawk system, Motion Analysis Inc.). The motion capture system included eight cameras with a sampling rate of 200 Hz and tracked forty-one passive retro-reflective markers. The tennis player was filmed standing at the middle of the baseline on the court (i.e., center mark position) and was asked to hit the ball with maximum effort toward two square targets on the opposite side of the court. The two target areas (1.5 m × 1.5 m) were set on the left side of the court (i.e., inside-out stroke) and on the right side of the court (i.e., cross-court stroke). A total of 12 successful shots, 6 inside-out, and 6 cross-court strokes, were used for motion capture data in CG animations. The positions of 21 anatomical landmarks on the body and 5 locations on the racket and ball were tracked during each trial (see details in Fukuhara et al., 2017).

Second, a CG tennis avatar (e.g., Ida et al., 2012; Fukuhara et al., 2017) was constructed from the motion capture data using character animation software (MotionBuilder 2013, Autodesk Inc.). The character modeling and AVI exporting were conducted with 3DCG software (Maya 2013, Autodesk Inc.). Moreover, a black background image that is traditionally used in biological motion perception studies was included in the CG animations (Johansson, 1973). The viewpoint was matched to the viewing angle of a receiver positioned at the midpoint of the service line on the tennis court. A tennis net was also inserted into the CG animations as a perceptual judgment criterion for the recognition task of the kinematic position. Here, we set a center strap in the net as a criterion point in the display. Additionally, the net mesh was deleted to avoid using another judgment criterion.

Third, the CG animations were set to four replay speeds to investigate the perceptual effects of the use of slow-motion footage: normal speed and three slow-speed motion conditions (three-quarter, half, and quarter speeds); the criterion used was previously described in a study on action observation with TMS (Moriuchi et al., 2014) and two studies on sports (Lorains et al., 2013; Uchida et al., 2014). Moreover, the length of the CG animations was set to 1,800 ms from the ready position to one frame (30 ms) before the moment of racket and ball contact. This occlusion point was adopted to avoid learning effects through feedback information because the moment of racket and ball contact slightly includes ball flight information after contact (Jackson and Mogan, 2007; Fukuhara et al., 2009).The replay duration for each of the four clips was 1,800, 2,400, 3,600, and 7,200 ms. In total, we created 48 video clips for analysis: 12 shots × 4 types of replay speeds.

### Procedure and Apparatus

Participants sat on a chair with their heads fixed on a chin support. The visual stimuli were presented on a 27-inch display monitor (GW2270HM-UN, BenQ, Taiwan; 1920 × 1080 resolution) connected to a laptop computer (ProBook450G2, HP, United States), and positioned at 0.5 m in front of participants. The vertical visual angle was approximately 20 degrees. Presentation software (E-prime 2.0, Psychological Software Tools Inc., United States) was used for visual stimuli and collection of participant responses.

**FIGURE 1:**
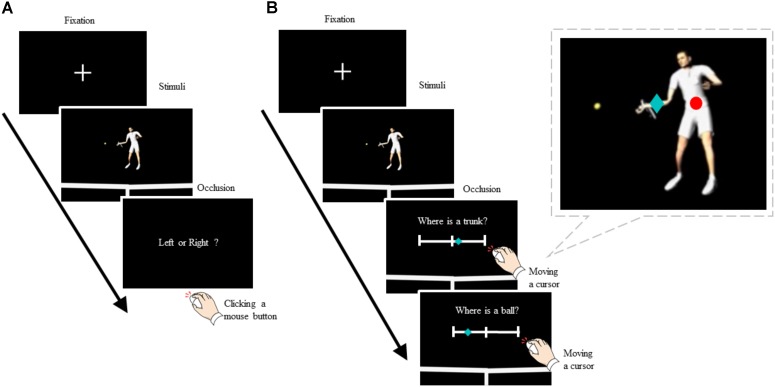
Experimental Settings. **(A)** Anticipatory judgment task. Participants were asked to click a mouse button with the right index finger (left side) or a middle finger (right side) to indicate the anticipated direction of the ball. **(B)** Recognition task of kinematic position. Participants were asked to evaluate the kinematic positions of trunk-center and ball in the CG avatar by moving a cursor with an optical mouse on a visual analog scale (VAS) as a reference to the center position, i.e., a center strap (VAS = 0 ± 0 m). The recognition errors were computed as the absolute value of the distance between the VAS score evaluated by the participants (transformed into the coordinate position in real scale, green rhombus) and the original coordinate position output from the motion capture data (red circle). A score change of 1 in the VAS score was equivalent to 2.9 cm in a real scale.

Two perceptual judgment tasks are shown in **Figure 1**. We decided to conduct these separately in this experiment to prevent a dual task involving attention to both tasks at the same time. An anticipatory judgment task was performed as the first block, and the recognition task of kinematic position was then performed as the second block. A total session was approximately 60 min (i.e., 30-min anticipatory judgment task, 30-min recognition task) in duration.

### Anticipatory Judgment Task

The participants were instructed to watch the visual stimulus presented and to anticipate the shot direction (left or right) (**Figure 1A**). We did not set a time constraint for responding but asked the participants to respond as soon as the stimulus was occluded by clicking the corresponding mouse buttons for the left and right targets. Prior to testing, the participants completed eight practice trials (four left and four right shot trials, which were randomly presented) to familiarize themselves with the task procedure. The practice trials included four different replay speeds. For the testing session, the participants completed 48 trials, and the stimuli were randomized.

### Recognition Task of Kinematic Position

Participants were instructed to evaluate the kinematic positions of the trunk-center and ball in the CG avatar immediately after observing the presented visual stimuli (**Figure 1B**). The visual stimuli were the same as those in the anticipatory judgment task, and the evaluation of position was performed only in the transverse direction (parallel to the net). The recognition of kinematic position was rated on the VAS by moving a computer mouse pointer over a slider bar, from -50 (left, equivalent to -1.45 m in real scale) to + 50 (right, + 1.45 m) in reference to the center position, i.e., a center strap (VAS = 0 ± 0 m). Participants first evaluated the position of the trunk-center and then the position of the ball.

Prior to testing, the participants completed eight practice trials (four left and four right shot trials, which were randomly presented) to familiarize themselves with the task procedure. The practice trials also included four different replay speeds. For the testing session, the participants also completed 48 trials, and the stimuli were randomized.

### Data Analysis

#### Correct Responses

The dependent variable was the percentage of correct responses for shot directions at each replay speed. All variables were converted to arcsine transformation to satisfy the normal distribution assumption. We evaluated data using a two-way factorial analysis of variance (ANOVA), with the two groups (skilled and novice) used as between-participants factors, and four replay speeds (normal-, three-quarters-, half-, and quarter-speed) as the within-participants factors. To investigate whether the percentage of correct responses exceeded a 50% guess level (chance level), one-sample *t*-tests were also performed to evaluate the percentage of correct responses in each experimental condition.

#### Recognition Errors

The dependent variable was the recognition error (cm) for two kinematic positions of the trunk-center and ball in the CG avatar at each replay speed. Fukuhara et al. (2017) examined kinematic cues for effective anticipation of shot directions by skilled tennis players using manipulation of graphical information richness in a CG avatar. Results suggested that skilled players used the movements of proximal (i.e., trunk, hips, and shoulders) and distal (i.e., racket-arm and ball) body parts to anticipate the direction of forthcoming shots, while novice players mainly focused on the movement of distal body parts (Ward et al., 2002; Huys et al., 2009; Williams et al., 2009). Based on these finding, we selected the trunk-center and ball as the evaluation items in a recognition task of kinematic position.

The VAS score ranging from -50 to +50 was equivalent to 2.90 m (from -1.45 m to +1.45 m) in the transverse direction; thus, a score change of 1 in the VAS was equivalent to a difference of 2.90 m/100 = 2.9 cm in real scale. The recognition errors were computed as the absolute value of the distance between the transformed VAS position and the original kinematic position obtained as the coordinate value of motion capture data (see **Figure 1B**). In each kinematic position (trunk-center and ball), two-way ANOVA was performed, with the 2 groups (skilled and novice) used as between-participants factors, and four replay speeds (normal-, three-quarters-, half-, and quarter-speed) as within-participants factors.

#### Correlation Between Correct Responses and Recognition Errors

Pearson’s correlation coefficient was computed between correct responses and recognition errors for two kinematic positions (trunk-center and ball) for each of the four replay speeds (normal-, three-quarters-, half-, and quarter-speed) in two group (skilled and novice) to investigate whether recognition of kinematic position has an influence on anticipatory judgments.

Bonferroni’s *post hoc* test for multiple comparisons was used for further analysis. Partial eta-squared (ηp^2^) values provided a measure of effect size. In all analyses, the significance level was set at α = 0.05.

## Results

### Correct Responses

**FIGURE 2:**
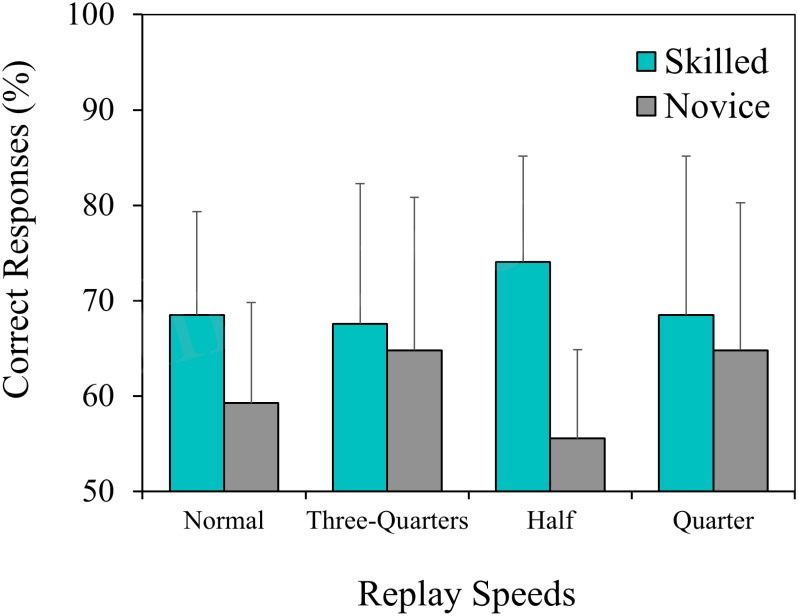
The mean accuracy score (skilled and novice groups) for each of the four replay conditions, with the chance level set at 50%.

The mean percentages of correct responses for skilled and novice groups are shown in **Figure 2**. The correct responses in the skilled group were significantly over chance levels of 50% (all *p* < 0.05), while the novice group was also significantly superior to chance levels (all *p* < 0.05), with the exception of the half-speed condition (*p* = 0.11).

Two-way ANOVA revealed a significant main effect for group [F (1,16) = 6.37, *p* < 0.05, $\eta_{p}^{2}$ = 0.29]: skilled players (*M* = 69.68%, *SD* = 12.14) showed more accurate performance than their novice counterparts (*M* = 61.11%, *SD* = 13.21). However, the main effect for replay speeds [F (3,48) = 0.28, *p* = 0.84, $\eta_{p}^{2}$ = 0.02] and the group × replay speed interactions [F (3,48) = 1.42, *p* = 0.25, $\eta_{p}^{2}$ = 0.08] were not significant.

### Recognition Errors

**FIGURE 3:**
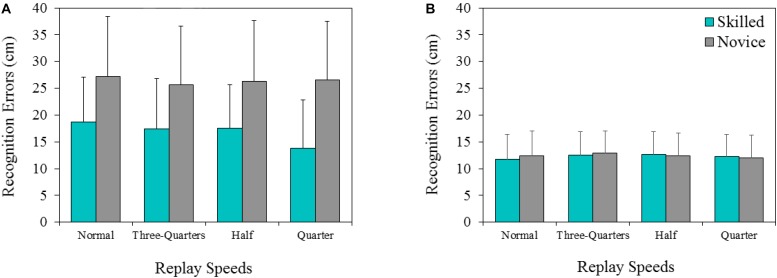
The mean error score (skilled and novice groups) for two evaluation positions **(A)**: trunk-center and **(B)**: ball) for each of the four replay conditions.

The mean percentages of recognition errors under each experimental condition for the skilled and novice groups are shown in **Figure 3**. A two-way ANOVA for the trunk-center condition (**Figure 3A**) identified a significant main effect for the replay speeds [F (3,48) = 3.41, *p* < 0.05, $\eta_{p}^{2}$ = 0.18], but *post-hoc* analyses indicated that there were no significant differences. The main effect of group was significant [F (1,16) = 4.02, *p* < 0.05, $\eta_{p}^{2}$ = 0.21]. *Post hoc* analysis indicated that recognition errors (16.85 cm) in the skilled group were smaller than those of their novice counterparts (26.43 cm) (*p* < 0.05). A group × speed interaction was significant [F (3,48) = 3.21, *p* < 0.05, $\eta_{p}^{2}$ = 0.17], indicating that the recognition errors of trunk-center in the skilled group for the quarter-speed condition were smaller than for all other speed conditions (all *p* < 0.05), while the novice group did not show any significant differences for replay speed. For the quarter-speed condition, skilled players were significantly more accurate than their novice counterparts (*p* < 0.05). On the other hand, a two-way ANOVA for the ball condition (**Figure 3B**) showed no significant main effect for group [F (1,16) = 0.02, *p* = 0.96, $\eta_{p}^{2}$ = 0.01] and replay speeds [F (3,48) = 0.55, *p* = 0.65, $\eta_{p}^{2}$ = 0.03]. There was no significant interaction for group × replay speeds (F (3, 48) = 0.57, *p* = 0.87, $\eta_{p}^{2}$ = 0.03).

### Correlations Between Correct Responses and Recognition Errors

In all experimental conditions, no significant correlation was observed between the two dependent variables (see **Supplementary Table 1** in the **Supplementary Materials**).

## Discussion

The present study investigated the effects on anticipatory judgment of shot directions and recognition of opponents’ kinematic positions (trunk center and ball) when skilled and novice tennis players viewed CG tennis shots at four different replay speeds (normal, quarter-half, half, and quarter speeds). We failed to show an immediate effect of the use of slow-motion footage on the anticipatory judgments of both the skilled and novice players. The correct responses in both skilled and novice players did not improve as the replay speeds decreased. In contrast to the results of the anticipatory judgments, we found reduced recognition errors regarding the trunk center position in the skilled players. The recognition errors in the trunk center position significantly improved in the slowest replay condition (quarter speed) compared with the other speed conditions. In the same condition, the skilled players more accurately recognized the trunk center position than their novice counterparts. Moreover, no significant correlation was observed between the anticipatory judgments and motion recognition in all experimental conditions. These results showed that extension of the observation time with slow-motion footage provided an added benefit of immediately enhancing the motion recognition by skilled players but did not improve the participants’ anticipatory judgments.

Before discussing the main finding regarding the immediate effects of using slow-motion footage, it is necessary to confirm whether our unique CG animations were valid in investigating anticipatory skills in tennis. The results showed that (i) the correct responses of both skilled and novice players were superior to chance levels of 50% (except for the novice players at the half-speed condition), and (ii) the anticipatory performance of the skilled players was superior to that of their novice counterparts. These results indicate that skilled players can pick up key kinematic cues from the CG avatar for effective anticipation when compared with their novice counterparts. These results are comparable with those of previous studies that used videos (Williams et al., 2002; Jackson and Mogan, 2007), point-light or stick figure displays (Ward et al., 2002; Huys et al., 2009; Williams et al., 2009), and CG animations (Fukuhara et al., 2009; Ida et al., 2012; Fukuhara et al., 2017). From these findings, we can safely say that our CG animations have sufficient quality for evaluating anticipatory skills in tennis.

The present findings did not support our hypothesis that the correct responses in both skilled and novice players would be improved with the decline in replay speeds, given that the forehand stroke in tennis is a rapid movement. This finding is inconsistent with previous findings, which showed that a slow-speed replay of observed actions (particularly rapid movements) enhanced the understanding of an opponent’s action intention when compared with a normal-speed replay (Moriuchi et al., 2014; Moriuchi et al., 2017). Moriuchi et al. (2017) have reported that the M1 excitability was higher only when observing low-speed replay videos of rapid movements than when observing normal-speed replay videos; however, the effect was not confirmed when viewing slow movements. The authors explained that the discrepancy between the two movement tasks was attributed to whether participants were able to acquire new information on kinematic elements that cannot be observed at normal speed; if individuals can recognize the kinematic elements at normal speed, the benefit of using slow-motion footage is not evident. In the present study, the one-sample *t*-test showed that both skilled and novice players were able to pick up kinematic cues from the CG avatar for anticipation of shot directions even under the normal-speed condition. Based on these findings, the failure to show the benefit of using slow-motion footage in the present study can be explained by the participants’ ability to recognize their opponents’ forehand stroke at normal speed.

The present findings are consistent with those of a previous study, which showed that there were no improvements in a video-based decision-making task in Australian football under the slow-speed condition (Lorains et al., 2013). Lorains et al. (2013) have clarified that the decision-making of elite footballers was more accurate in the fast-speed video (1.5-times faster speed) than in the normal- and slow- (0.75 times) speed videos. The authors suggested that the time pressure in the speeded video may allow elite footballers to perform more automatic processing required in an actual game situation. Considering this, skilled anticipation may not be sufficiently aided by the use of slow-motion footage without severe time constraints (i.e., time pressure).

In contrast to the findings of the anticipatory judgments, the present findings partially supported our hypothesis that the recognition errors in both skilled and novice players would be improved with the decline in replay speeds. We found that slow-motion footage has a perceptual feature that immediately enhances the motion recognition of the trunk position by skilled players. Previous studies on tennis (Huys et al., 2009; Williams et al., 2009) have reported that skilled tennis players used the movements of the proximal (i.e., trunk, hips, and shoulders) and distal (i.e., racket-arm and ball) body parts of an opponent to anticipate shot directions, whereas novice players mainly focused on distal body information. More recently, Fukuhara et al. (2017) have suggested that the role of using proximal body information among skilled players may be to anticipate subsequent movements of distal body parts. Such visual attention was also reported in another study (Piras et al., 2015) that investigated microsaccades when elite table tennis players anticipated shot directions. Moreover, in the present study, the recognition errors by the novice players were not significantly different among the four replay conditions; this indicated that the use of slow-motion footage did not provide an added benefit of immediately enhancing the recognition of the opponents’ kinematic position among the novice players. Considering these findings, the skilled players, but not the novice players, may have qualitatively developed a specific motion recognition ability to recognize the movements of proximal body parts accurately.

Contrary to our expectation, there were no correlations between the anticipatory judgments and recognition of kinematic positions in all experimental conditions. The enhancement of position recognition induced with the use of slow-motion footage had no influence on the anticipatory judgment. This finding indicates that even if recognition of a specific kinematic feature (i.e., position or orientation of the trunk) is facilitated by the use of slow-motion footage, such information pick-up might not be effective for successful anticipation.

This study has some limitations. First, we investigated and classified nine elite college tennis players (members of the champion teams of Japan intercollege tournaments in 2016) into the skilled group; there were nine players in each of the skilled and novice groups. However, the number of participants was relatively smaller than those in previous studies on racket sports (Lorains et al., 2013; Schweizer and Furley, 2016). Thus, it is necessary to examine this issue further using larger sample sizes.

Second, the present study aimed to conduct the anticipatory judgment task and the recognition task separately. This may be one reason why there were no significant correlations between the anticipatory judgments and the recognition of kinematic positions. The reason for separating the two tasks was to prevent a dual task involving paying attention to both tasks at the same time. However, by separating both tasks, we might not directly evaluate the recognition of the kinematic positions during anticipation of the shot directions. Future studies are needed to investigate whether there is a relationship between the two dependent values when the tasks are performed simultaneously.

Third, the research method regarding the recognition task of kinematic positions may have affected our results. The participants evaluated the two kinematic positions (trunk center and ball) only on the horizontal axis of the display. However, some anticipation studies on tennis have reported that skilled tennis players used not only the movements of the trunk but also those of other proximal body parts (e.g., shoulders, hips, and legs) for anticipating shot directions (Huys et al., 2009; Williams et al., 2009). Considering this, the results of the present study might reflect only a part of the motion recognition ability of skilled players. In future studies, it is necessary to use a novel evaluation method that can assess high-resolution spatial information, such as touch panel computer (two-dimensional space) or virtual reality environment (three-dimensional space). If such evaluation methods are established, we would be able to investigate the degree to which skilled players accurately recognize the entire body movements of an opponent in detail.

## Conclusion

The aim of the present study was to clarify whether the use of slow-motion footage of forehand strokes can immediately improve anticipatory judgments of shot directions and recognition of kinematic positions in tennis. We failed to show the immediate effects of the use of slow-motion footage on the anticipatory judgments of the skilled and novice players. Instead, we found that slow-motion footage has a perceptual feature that immediately enhances the fine-tuning of recognition of the trunk position by skilled players. Moreover, no significant correlation was observed between the anticipatory judgments and motion recognition in all experimental conditions. From these results, we concluded that even if the recognition of opponents’ kinematic cues is facilitated by the use of slow-motion footage, such information pick-up might not be effective for immediately improving the anticipatory judgments in tennis.

## Author Contributions

KF and TM designed and conducted an experiments. KF wrote the manuscript. TO, BS, and MI helped planning our experimental paradigm. TH and HI supervised this study.

## Conflict of Interest Statement

The authors declare that the research was conducted in the absence of any commercial or financial relationships that could be construed as a potential conflict of interest.
