# Supplementary material for: Can Slow-Motion Footage of Forehand Strokes Be Used to Immediately Improve Anticipatory Judgments in Tennis?
Source: Front Psychol. 2018 Oct 4;9:1830. doi: 10.3389/fpsyg.2018.01830 (PMC6180172; doi:10.3389/fpsyg.2018.01830)
Supplement: Supplementary file 1 [file Table_1.PDF]

Supplementary Material

Can slow-motion footage of forehand strokes be used to immediately improve anticipatory judgments in tennis?

Kazunobu Fukuhara*, Tomoko Maruyama, Hirofumi Ida, Takahiro Ogata, Bumpei Sato, Motonobu Ishii, and Takahiro Higuchi

*** Correspondence:** Kazunobu Fukuhara: fukuhara-k@tmu.ac.jp

1 Supplementary Tables

Supplementary Table 1. The statistical data (r and p-value) of Person's correlation coefficient between correct responses and recognition errors for two kinematic positions (trunk-center and ball) for each of the four replay conditions.

Group	Replay Speed	Kinematic Position	*r*	*p*
Skilled	Normal	Trunk-center	-0.209	0.590
		Ball	0.411	0.234
	Quarter-half	Trunk-center	-0.399	0.287
		Ball	0.006	0.989
	Half	Trunk-center	0.392	0.296
		Ball	-0.213	0.582
	Quarter	Trunk-center	0.235	0.543
		Ball	0.394	0.294
Novice	Normal	Trunk-center	-0.393	0.296
		Ball	-0.228	0.556
	Quarter-half	Trunk-center	-0.022	0.955
		Ball	-0.291	0.447
	Half	Trunk-center	-0.140	0.719
		Ball	-0.210	0.588
	Quarter	Trunk-center	-0.027	0.945
		Ball	-0.200	0.605
